# Aspergilloma Coexisting With Idiopathic Pulmonary Fibrosis: A Rare Clinical Entity

**DOI:** 10.7759/cureus.59323

**Published:** 2024-04-29

**Authors:** KK Athish, TJ Guruprasad, Spurthy Padmanabha, Harshitha KR

**Affiliations:** 1 Internal Medicine, Sri Devaraj Urs Academy of Higher Education and Research, Kolar, IND; 2 Respiratory Medicine, Sri Devaraj Urs Academy of Higher Education and Research, Kolar, IND; 3 Pulmonology, Sri Devaraj Urs Academy of Higher Education and Research, Kolar, IND

**Keywords:** interstitial lung disease, usual interstitial pneumonia (uip), traction bronchiectasis, cystic bronchiectasis, idiopathic pulmonary fibrosis, pulmonary aspergilloma

## Abstract

Aspergilloma also known as fungal ball or mycetoma, is a saprophytic mycotic infection caused by *Aspergillus* species which usually colonizes pre-existing cavitary or cystic lesions in the lung. Here, we have a rare case of idiopathic pulmonary fibrosis (IPF) with bilateral bronchiectasis complicated by aspergilloma. Although the existence of aspergilloma is common in pre-existing lung cavities, its coexistence in patients with IPF is a rarity, and the incidence of such cases in the literature remains sparse. Here is an interesting case report of aspergilloma co-existing with IPF. This article comprehensively analyzes the existing literature depicting similar associations and the possible etiology for the development of aspergilloma in patients with IPF.

## Introduction

Aspergillosis is a ubiquitous, airborne mycotic disease caused by *Aspergillus*, a filamentous, saprophytic fungus. Aspergilloma is a subtype of chronic pulmonary aspergillosis. Inhaled conidia of *Aspergillus *adhere to the pre-existing cavity walls in the lung parenchyma. However, pre-existing airway pathologies and chronic cavitary lung lesions predispose the development of aspergilloma in immunocompromised hosts due to defective immune responses [1,2]. Idiopathic pulmonary fibrosis (IPF) is a long-standing progressive irreversible fibrotic interstitial lung pathology with unclear etiology, which typically reveals a usual interstitial pneumonia (UIP) pattern histologically [3]. Here is a case of the left upper lobe cavity, with aspergilloma, bilateral bronchiectasis, and radiological features suggestive of UIP, that is, IPF. To our knowledge, our case report is the first to describe these associations.

## Case presentation

**Figure 1:**
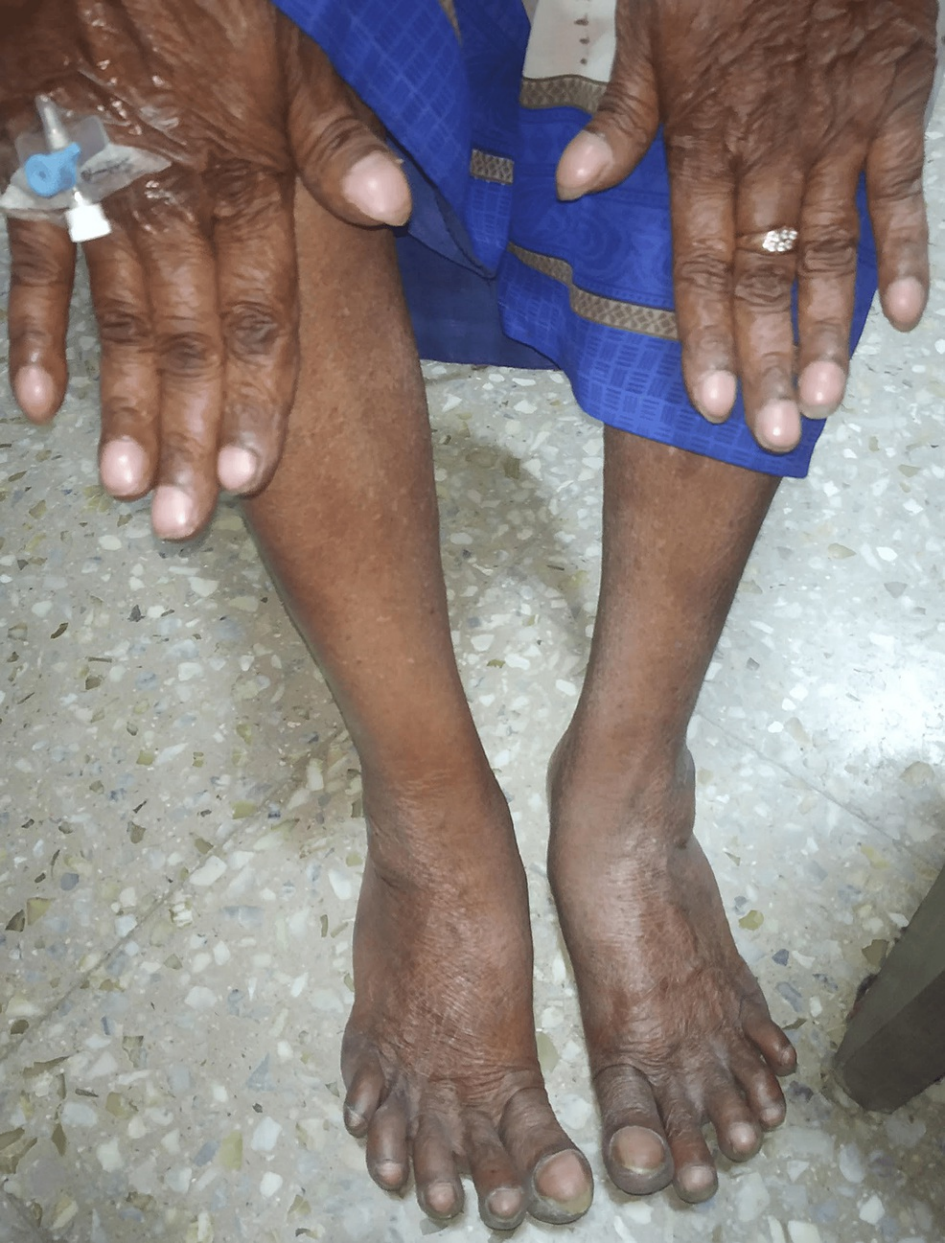
Grade III clubbing in all the digits of bilateral upper and lower limbs.

A 76-year-old Indian female presented with concerns of dry cough and progressive shortness of breath of six months duration and denied other complaints. She has been on oral hypoglycemic agents for type 2 diabetes mellitus for 15 years. There was no history of pulmonary tuberculosis. There was no history of exposure to pets, molds, asbestos, silica, or tobacco. Examination revealed a respiratory rate of 21 cycles per minute, at room air oxygen saturation was 95%, afebrile and grade III clubbing (Figure 1). The rest of the general physical examinations were unremarkable.

**Figure 2:**
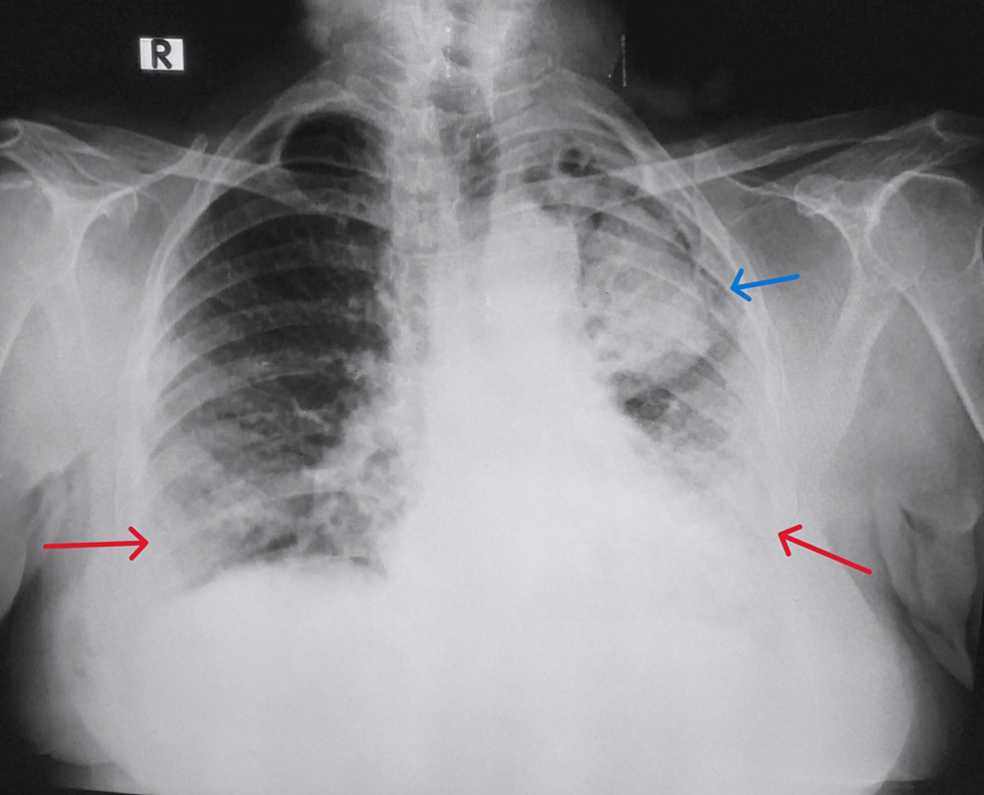
Chest radiograph revealed a tracheal shift to the left, a cavity in the left upper and mid-zone with intracavitary space-occupying lesion (blue arrow), bilateral mid and lower zone non-homogenous reticulonodular opacities with haziness (red arrows), and crowding of ribs were noted.

Respiratory system examination revealed left-sided rib crowding and bronchial breath sounds in the left infraclavicular and infra-axillary areas. Left infraclavicular and axillary mid-inspiratory coarse crepitations and bilateral lower zone end-inspiratory fine crepitations were heard on auscultation. The rest of the systemic examination was unremarkable. Arterial blood gas (ABG) analysis revealed hypoxemic respiratory failure. Other laboratory parameters were within normal limits. The chest radiograph revealed a cavity in the left upper lobe with an intracavitary lesion and bilateral lower zone reticular opacities (Figure 2).

The electrocardiogram and cardiac ultrasound (2D ECHO) were normal. Antinuclear antibodies (ANA), anti-double stranded DNA (anti dsDNA), anti-neutrophil cytoplasmic antibodies (ANCA), rheumatoid factor (RA), anti-cyclic citrullinated peptide (Anti-CCP), and other rheumatologic tests were negative. Interferon-gamma release assay (IGRA) revealed a negative test result (Table 1).

**Table 1:** Serology profile.

Serological tests	Result
Antinuclear antibodies (ANA) (immunofluorescence)	Negative
Anti-double stranded DNA (anti dsDNA)	Negative
Anti-neutrophil cytoplasmic antibodies (ANCA)	Negative
Rheumatoid factor (RA)	Negative
Anti-cyclic citrullinated peptide (anti-CCP)	Negative
Interferon-gamma release assay (IGRA)	Negative

Bronchoscopy and bronchoalveolar lavage (BAL) were performed. The mucosa was normal with no obvious endobronchial lesion. BAL fluid cytology revealed the presence of alveolar macrophages, neutrophils, and lymphocytes within the normal range (Table 2). BAL fluid gram stain revealed no organisms, and the potassium hydroxide (KOH) mount revealed fungal elements. BAL fluid aerobic bacterial culture revealed no growth of organisms, however, fungal culture revealed growth of *Aspergillus fumigatus*. A cartridge-based nucleic acid amplification test (CBNAAT) did not detect *Mycobacterium tuberculosis* in BAL fluid. During the six-minute walk test (6MWT), a fall in oxygen saturation to 89% from the baseline resting saturation of 95% was noted after the patient walked a distance of 240 meters. Spirometry revealed a mixed pattern with mild obstruction and moderate restriction with no bronchodilator reversibility. The diffusing capacity of the lung for carbon monoxide (DLCO) was reduced.

**Table 2:** Bronchoalveolar lavage fractional cell analysis.

Parameters	Values
Return volume	50 mL
Recovery percentage	50%
Alveolar macrophages	90%
Lymphocytes	5%
Polymorphonuclear neutrophils	2%
Eosinophils	< 1%
Mast cells	< 1%
Atypical cells	Nil

**Figure 3:**
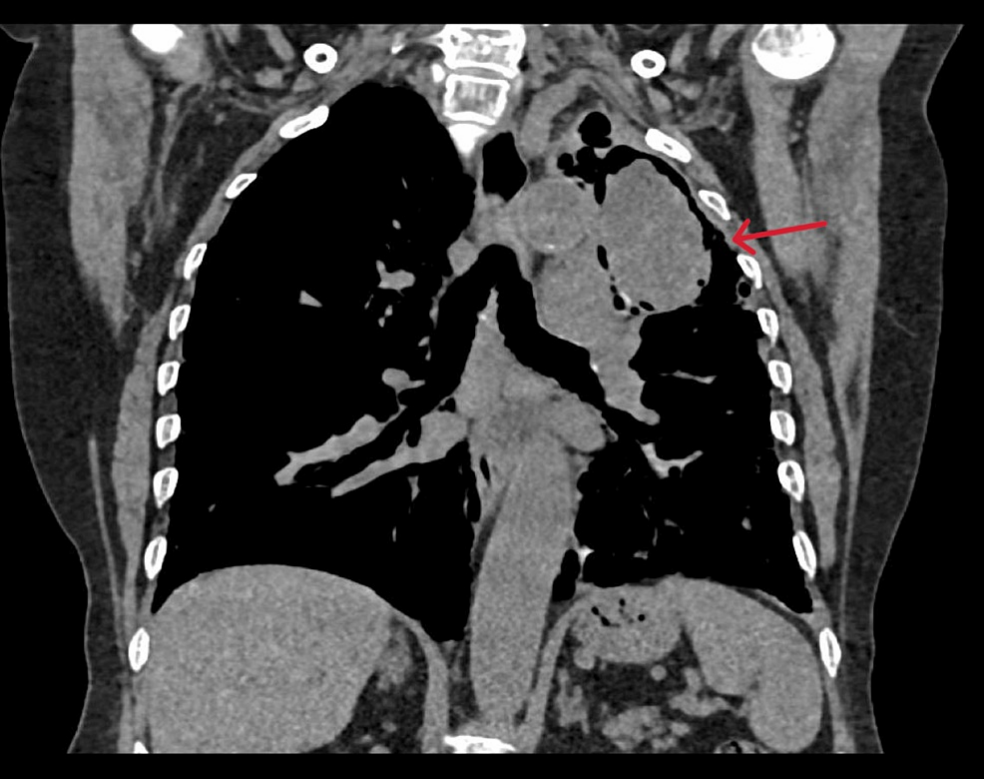
Computed tomography of the thorax-mediastinal window; coronal section showing a large tissue density lesion in the left upper lobe.

**Figure 4:**
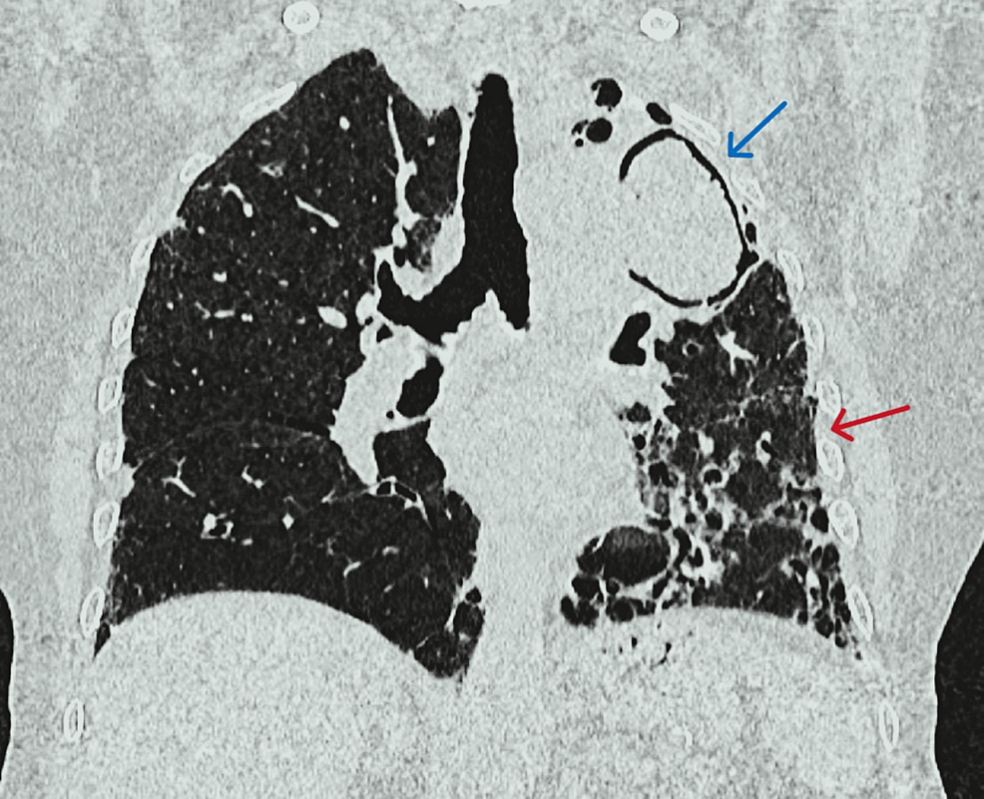
High-resolution computed tomography (HRCT) thorax showed a large cavity with an intracavitary fungal ball (blue arrow) in apicoposterior segment of the left upper lobe (67×40×60 mm) with soft tissue density area within and adjacent areas of consolidation, fibrosis and traction bronchiectasis (red arrow)in the left upper lobe.

**Figure 5:**
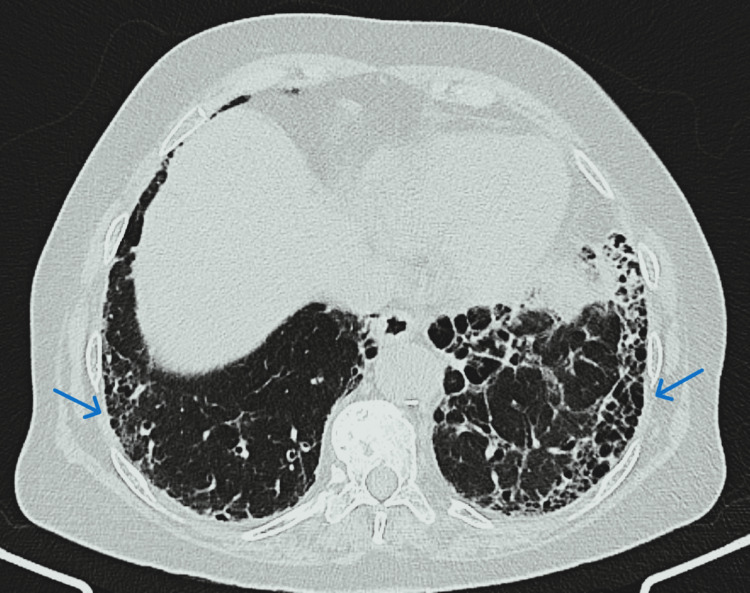
High-resolution computed tomography (HRCT) thorax revealed multiple areas of honeycombing involving bilateral lower lobes (left > right), and basal subpleural areas of fibrosis (likely suggestive of interstitial lung disease) consistent with definite UIP pattern.

On reviewing with clinical presentation, high-resolution computed tomography (HRCT) thorax findings (Figures 3-5), and exclusion of other possible causes for interstitial lung disease (ILD) with UIP pattern like connective tissue disorder, pneumoconiosis, hypersensitivity pneumonitis, radiation/drug-induced ILD, the patient was diagnosed as a case of aspergilloma co-existing with IPF. The patient was treated with itraconazole and pirfenidone. Besides antifungals and antifibrotics, long-term oxygen therapy, and nebulized bronchodilators were advised for symptomatic relief. The patient was reviewed on an outpatient basis after one month of therapy and was symptomatically better.

## Discussion

Aspergillus is known to cause a wide range of lung pathologies which are greatly influenced not only by the host’s immune status but also by the underlying structural lung abnormality from a simple colonization in the respiratory tract to hypersensitivity reactions such as allergic bronchopulmonary aspergillosis (ABPA), hypersensitivity pneumonitis, aspergilloma; or invasive pulmonary aspergillosis [4,5]. Generally, predisposing factors like allergic conditions including bronchial asthma, airway diseases such as chronic obstructive pulmonary disease (COPD) and bronchiectasis, as well as chronic cavitating lung diseases namely tuberculosis, emphysema, sarcoidosis, and interstitial lung disease, or immunocompromised state, favors the development of pulmonary aspergillosis [1,2]. Indeed, any lung cavity could serve as a potential focus for aspergilloma.

Over 95% of aspergilloma cases in undeveloped countries are caused in patients with past evidence of tuberculosis, which is the most frequent cause of the disease. Structural aspects of established bronchiectasis further predispose to *Aspergillus* disease. The etiopathogenesis of aspergillosis in bronchiectasis is diverse. Studies have shown the association of aspergillosis with bronchiectasis to have worse clinical outcomes with increased rates of exacerbations leading to hospitalization, particularly in elderly patients and in those receiving long-term antibiotic therapy [6].

Aspergillomas typically appear on radiographs as a circular mass inside a cavity with air surrounding the mass (crescent sign), which appears to gravitate freely in various positions [7]. Majority of aspergillomas remain stable in size, however, 10% of cases result in spontaneous resolution or a size reduction, correlating the arrest of expansion with successful therapy. Less frequently aspergillomas show an increase in size [8]. In patients with past evidence of pulmonary tuberculosis, the upper lobes of the lungs are the most common location of aspergilloma. However, its association with IPF is mentioned in fewer case reports.

IPF is a long-standing, progressive irreversible ILD of unclear etiology presenting as UIP secondary to chronic lung fibrosis. Recently, epithelial diseases caused by recurring micro-injuries, and defective type II alveolar epithelial cells (ATII) which are incapable of supporting normal lung regeneration, have been linked to the pathophysiology of IPF. Furthermore, a pathological tendency towards fibrosis rather than regeneration is caused by an abnormal interaction between epithelial and mesenchymal cells. Type II alveolar cell damage is mostly caused by genetic predispositions, aging, and environmental variables. As IPF is a form of fibrosing interstitial pneumonia, patients usually present with exertional dyspnea, dry cough, and inspiratory crackles on auscultation. Antifibrotic medications namely pirfenidone and nintedanib, are an effective treatment for IPF [8].

There are only two cases of aspergilloma and IPF mentioned in the literature that we were able to come across [9,10]. There is no recognized mechanism for aspergilloma colonization in UIP. According to Kumar et al, it might be related to the use of immunosuppressants in IPF treatment [9]. Similarly, rarely do aspergilloma and UIP coexist. When a UIP/ILD patient exhibits hemoptysis, aspergilloma should be suspected and looked into. In immunocompetent patients, the development of aspergillus lung disease may be related to the presence of established honeycomb structures and traction bronchiectasis brought on by excessive fibrotic processes, a feature consistent with chronic IPF which is not a cavitary disease [10].

## Conclusions

Although the occurrence of aspergilloma in preexisting cavitary lesions is common, its association with idiopathic pulmonary fibrosis (IPF) is not proven. We therefore report a rare case of aspergilloma co-existing with IPF. This study highlights the intricate relationship between aspergilloma and complex lung pathologies like IPF. The rare coexistence of aspergilloma with IPF, as demonstrated in our case, underscores the need for a high degree of suspicion and individualized treatment approaches. Our findings advocate for continued vigilance in the management of IPF patients, particularly when immunosuppressants are involved. This case contributes to the limited literature on aspergilloma in the context of IPF, paving the way for further research into these challenging and multifaceted clinical scenarios.
